# CD8^+^PD-L1^+^CXCR3^+^ polyfunctional T cell abundances are associated with survival in critical SARS-CoV-2–infected patients

**DOI:** 10.1172/jci.insight.151571

**Published:** 2021-09-22

**Authors:** Lucille Adam, Pierre Rosenbaum, Paul Quentric, Christophe Parizot, Olivia Bonduelle, Noëlline Guillou, Aurélien Corneau, Karim Dorgham, Makoto Miyara, Charles-Edouard Luyt, Amélie Guihot, Guy Gorochov, Christophe Combadière, Behazine Combadière

**Affiliations:** 1 Sorbonne University, Inserm U1135, Center for Immunology and Infectious Diseases, Cimi-Paris, Paris, France.; 2 Assistance Publique-Hôpitaux de Paris (AP-HP), Pitié-Salpêtrière University Hospital, Department of Immunology, Paris, France.; 3 Sorbonne University, UMS037, PASS, Pitié-Salpêtrière Cytometry Platform (CyPS), Paris, France.; 4 AP-HP, Pitié-Salpêtrière University Hospital, Department of Pulmonology, Intensive Medicine, and Resuscitation, Paris, France.; 5 Sorbonne University, Inserm, Institute of Cardiometabolism and Nutrition, Paris, France.

**Keywords:** Immunology, Adaptive immunity, T cells

## Abstract

The importance of the adaptive T cell response in the control and resolution of viral infection has been well established. However, the nature of T cell–mediated viral control mechanisms in life-threatening stages of COVID-19 has yet to be determined. The aim of the present study was to determine the function and phenotype of T cell populations associated with survival or death of patients with COVID-19 in intensive care as a result of phenotypic and functional profiling by mass cytometry. Increased frequencies of circulating, polyfunctional CD4^+^CXCR5^+^HLA-DR^+^ stem cell memory T cells (Tscms) and decreased proportions of granzyme B–expressing and perforin-expressing effector memory T cells were detected in recovered and deceased patients, respectively. The higher abundance of polyfunctional PD-L1^+^CXCR3^+^CD8^+^ effector T cells (Teffs), CXCR5^+^HLA-DR^+^ Tscms, and anti-nucleocapsid (anti-NC) cytokine-producing T cells permitted us to differentiate between recovered and deceased patients. The results from a principal component analysis show an imbalance in the T cell compartment that allowed for the separation of recovered and deceased patients. The paucity of circulating PD-L1^+^CXCR3^+^CD8^+^ Teffs and NC-specific CD8^+^ T cells accurately forecasts fatal disease outcome. This study provides insight into the nature of the T cell populations involved in the control of COVID-19 and therefore might impact T cell–based vaccine designs for this infectious disease.

## Graphical abstract

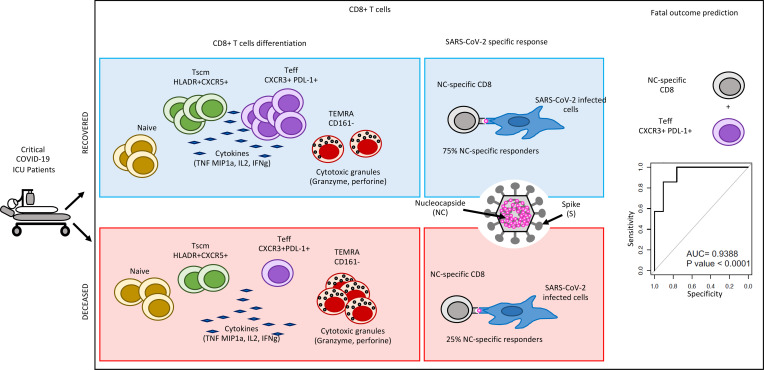

## Introduction

The emergence of the new SARS-CoV-2–induced coronavirus disease 2019 (COVID-19) outbreak has rapidly emerged as an important healthcare, societal, and economic threat due to its extremely fast worldwide spreading and severity. In January 2021, more than 88 million cases and 1.9 million deaths have been reported (https://covid19.who.int). Even if the vast majority of cases are asymptomatic or characterized by mild symptoms (1, 2), 6% of patients infected with SARS-CoV-2 suffer from severe symptoms after the development of acute respiratory distress syndrome (ARDS) that can lead to death. Those severe to critical cases have a fatality rate of 2%–8% and require an admission in an intensive care unit (ICU; ref. 3). During SARS-CoV-2 infection, numerous studies have pointed out a dysregulated and disruptive innate immune response inducing a general global hyperinflammation that leads to disease aggravation (4). This hyperinflammation has been associated with lung damage and ARDS with fatal outcomes. It is therefore reasonable to postulate that the inflammatory response measured at both the cellular and molecular levels could represent a strong prognostic signature of the disease. The dysregulation of innate cell function and a decreased production of type I and type III IFNs have been highlighted as key contributors to viral persistence and disease severity (5, 6). However, decades of works on antiviral T cell responses have underscored their essential role in viral clearance (7–9). Thus, we aimed to decipher T cell differentiation and functional profiles during critical SARS-CoV-2 infection in ICUs.

SARS-CoV-2–specific CD4^+^ T cell and CD8^+^ T cell responses have been described to be robustly induced in moderately to severely ill patients with COVID-19 (10–12). However, marked alterations in phenotypical and functional properties of SARS-CoV-2–specific T cells have been observed in severely ill patients compared with convalescent patients (12). During acute viral infection, viral peptides activate the naive T cell compartment, initiating proliferation and differentiation of T cells into effector and memory cell subsets that participate in effective viral clearance (13–17). During severe SARS-CoV-2 infection, virus-specific CD4^+^ T cells were found to be dominant over CD8^+^ T cells with marked Th1 polarized CD4^+^ T cells specific for the viral spike protein, although Th2 and Th17 cytokines were also detectable (18). Atypical T cell differentiation seems to occur in patients with COVID-19, with T cells partially resembling Th1, Th2, Th17, and T follicular helper (Tfh) but lacking their principal feature including hyperactivation observed in the most severely ill patients (19). In addition, single-cell analyses of virus-specific CD4^+^ T cells showed that their gene expression patterns were distinct with disease severities (20). Strong memory CD4^+^ T cells and CD8^+^ T cells were induced in convalescent individuals after COVID-19 disease, suggesting a role of T cell immunity in disease control (11); however, the type of response favoring recovery or symptoms worsening is yet to be determined.

For the past 2 decades, studies of T cell differentiation and functional markers have distinguished between naive (CD45RA^+^CCR7^+^), activated, and effector (Teff; secretion of cytokines and cytolytic molecules). A fraction of these cells survives as memory T cells that contribute to long-term immunity. These memory cells are subdivided into central memory (Tcm), effector memory (Tem), and stem cell memory (Tscm) T cell compartments, according to their phenotype CD45RA^–^CCR7^–^, CD45RA^–^CCR7^+^, and CD45RA^+^CCR7^+^CD95^+^, respectively (21, 22). In addition to surface markers defining the differentiation of naive into memory T cells, their homing capacities as well as the definition of exhaustion and inhibitory cell populations are changeable after acute and chronic infection (7, 22–24). Tems contribute to viral control, whereas tissue-homing capacities combined with cytotoxicity could induce tissue damage and accelerate mortality, and T cell–mediated viral clearance impedes disease exacerbation (25, 26). Thus, a disequilibrium among T cell subsets could be detrimental to viral control. During SARS-CoV-2 infection, a marked increase in terminally differentiated Tem cells (Temras) and Tems as well as the concomitant decrease in the frequency of naive CD8^+^ cells have been observed (18). SARS-CoV-2–specific CD4^+^ T cells displayed major Tcm phenotype, whereas SARS-CoV-2–specific CD8^+^ T cells are more heterogeneous with Tem, Temra (18), or Tscm and transitional memory phenotype (27). SARS-CoV-2–specific T cells are present relatively early and increase over time (18). Thus, it seems reasonable to hypothesize that the quantitative and functional alteration of the T cell compartment would affect disease outcome.

In our study, we used mass cytometry to decrypt phenotypic and functional profile of T cell responses from patients with SARS-CoV-2 admitted to ICUs with or without fatal outcome. Approximately 15 days after symptoms, we detected a higher abundance of multiple cytokine-producing CXCR5^+^HLA-DR^+^ among CD4^+^ T cells and nucleocapsid-specific (NC-specific) CD8^+^ T cells, CXCR5^+^HLA-DR^+^ Tscms, and PD-L1^+^CXCR3^+^CD8^+^ Teffs in patients who recovered from COVID-19 than in patients who died. The paucity of PD-L1^+^CXCR3^+^ Teffs and NC-specific CD8^+^ T cells was associated with a fatal disease outcome.

## Results

### Skewed CD4^+^ T cells in critical SARS-CoV-2 infection survival.

**Figure 1:**
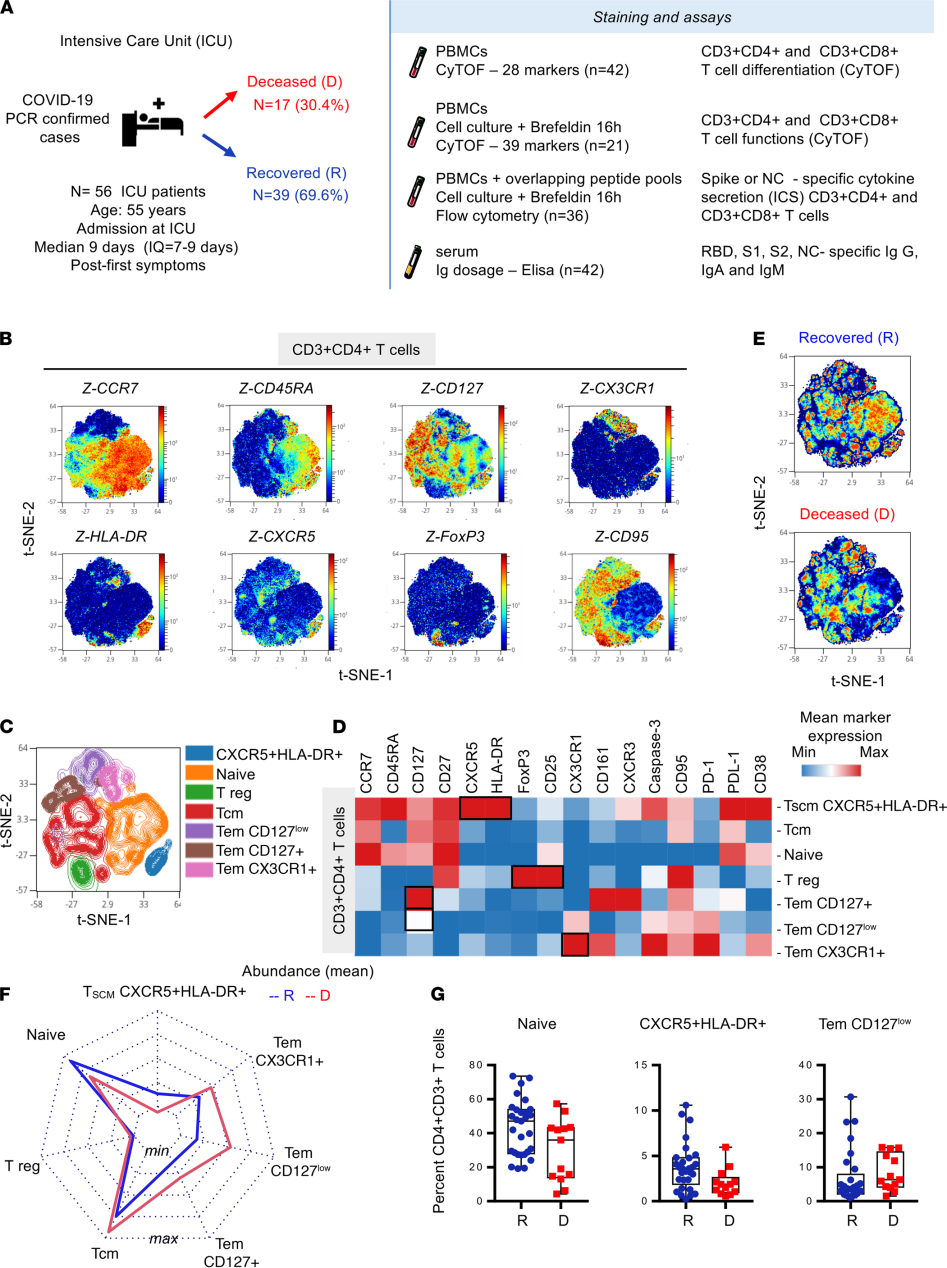
Increased CXCR5^+^HLA-DR^+^CD4^+^ T cells and decreased Tem subsets outline survival of critical cases with SARS-CoV-2 infection. (**A**) Fifty-six patients with confirmed SARS-CoV-2 infection were admitted in ICU at a median of 9 days after symptoms. PBMC samples were collected to assess T cell phenotypes (*n* = 42, R = 29, D = 13) and expression of effector molecules (*n* = 21, R = 12, D = 9) using mass cytometry panels 1 and panel 2 (Supplemental Tables 1 and 2, respectively). SARS-CoV-2 peptide–specific, cytokine-producing T cells were analyzed by flow cytometry (S1, S2 *n* = 46, R = 31 and D = 15; NC *n* = 39, R = 28 and D = 11). Humoral responses were measured in the serum (*n* = 42, R = 29, D = 13). CD3^+^CD4^+^ T cell (50,000 events) were randomly taken among sample for unsupervised cluster using FlowSOM. (**B**) Density plot t-SNE representing the expression of indicated markers. (**C**) Spatial t-SNE representing 7 major clusters as indicated. (**D**) Heatmap representation of mean signal intensity of each marker in identified CD3^+^CD4^+^T cell populations. (**E**) Density plot t-SNE representing abundance of events using concatenated files of 29 R and 13 D patients. (**F**) Radar representing mean (min/max normalized) abundance of CD3^+^CD4^+^T cell subsets in 29 R (blue) and 13 D (red) patients. (**G**) Box-and-whisker plots with min and max of CD3^+^CD4^+^T cell subset abundances in 29 R (blue) and 13 D (red) patients. All points are shown. Multiple Mann-Whitney *U* test using Benjamini, Krieger, and Yekutieli FDR correction was performed, with significance set at *q* < 0.05. R, recovered; D, deceased; Tem, effector memory T cell; tSNE, t-distributed stochastic neighbor embedding.

Fifty-six patients with confirmed SARS-CoV-2 infection were admitted to ICUs at a median of 9 days after symptoms (Table 1). To decipher the quality and intensity of adaptive immune responses in ICU patients who recovered and those who deceased, samples were analyzed for T cell responses as described in Figure 1A. Approximately one third of these patients deceased at a median of 21 days after ICU admission. Two CyTOF panels (Supplemental Tables 1 and 2; supplemental material available online with this article; https://doi.org/10.1172/jci.insight.151571DS1) were designed to study T cell phenotypes (*n* = 42) and the expression of effector molecules (*n* = 21). In addition, SARS-CoV-2 peptide-specific, cytokine-producing T cells were analyzed by flow cytometry (*n* = 36). Receptor binding domain (RBD), spike subunits 1 (S1) and 2 (S2), and NC-specific IgM, IgG, and IgA were also measured in the serum (*n* = 42).

Multiparametric mass cytometry staining helped to characterize CD4^+^ T cell subsets using unsupervised analysis on panel 1 of 28 surface markers in 42 ICU patients (Supplemental Table 1A). Visualization of t-distributed stochastic neighbor embedding (viSNE implementation of t-SNE algorithm) of CD3^+^CD4^+^ T cells (Figure 1B) using density plot revealed the distribution of several markers, including CCR7, CD45RA, CD127, CX3CR1, CXCR5, HLA-DR, FoxP3, and CD95, as depicted in Figure 1B. CCR7 and CD45RA mapping and indicated markers allowed us to label T cell subsets, according to the literature nomenclature, as naive, Tem, and Tcm (Supplemental Figure 1B). Seven major clusters were then defined and represented in t-SNE (Figure 1C). An additional heatmap (Figure 1D) allowed us to visualize the expression levels of 16 major surface markers. According to these results, among CD3^+^CD4^+^ T cells, we defined the following clusters: naive (CCR7^+^CD45RA^+^CD127^+^CD27^+^); CXCR5^+^HLA-DR^+^ T cells (CCR7^+^/intCD45RA^+^ also expressing CD27, CXCR3, and CD95 exhaustion markers PD-1 and PD-L1); Treg (expressing FoxP3 and CD25); Tem CD127^+^ (also expressing CX3CR1 and CD161); Tem CD127^low^; and Tem CX3CR1^+^ (also expressing CXCR3 and CD95). According to the literature, CXCR5^+^HLA-DR^+^ T cells might be Tscm because they share CCR7, CD45RA, CD27, CXCR3, and CD95 with Tscm, as previously described in patients with HIV (22). Visualization of concatenated files in density plot according to recovered and deceased patients showed significant differential abundance of these clusters (Figure 1E). As shown in the radar chart and box plots (Figure 1, F and G), we found that in deceased patients, naive and Tscm CXCR5^+^HLA-DR^+^ T cells were less abundant, whereas Tem CD127^lo^ were of higher frequencies, but not significant (multiple Mann-Whitney *U* test using Benjamini, Krieger, and Yekutieli FDR correction). These results reveal an unbalanced, but not statistically significant, CD4^+^ T cell differentiation among recovered and deceased patients.

### CD4^+^ T cell differentiation and functional profiles in critically infected patients with COVID-19.

**Figure 2:**
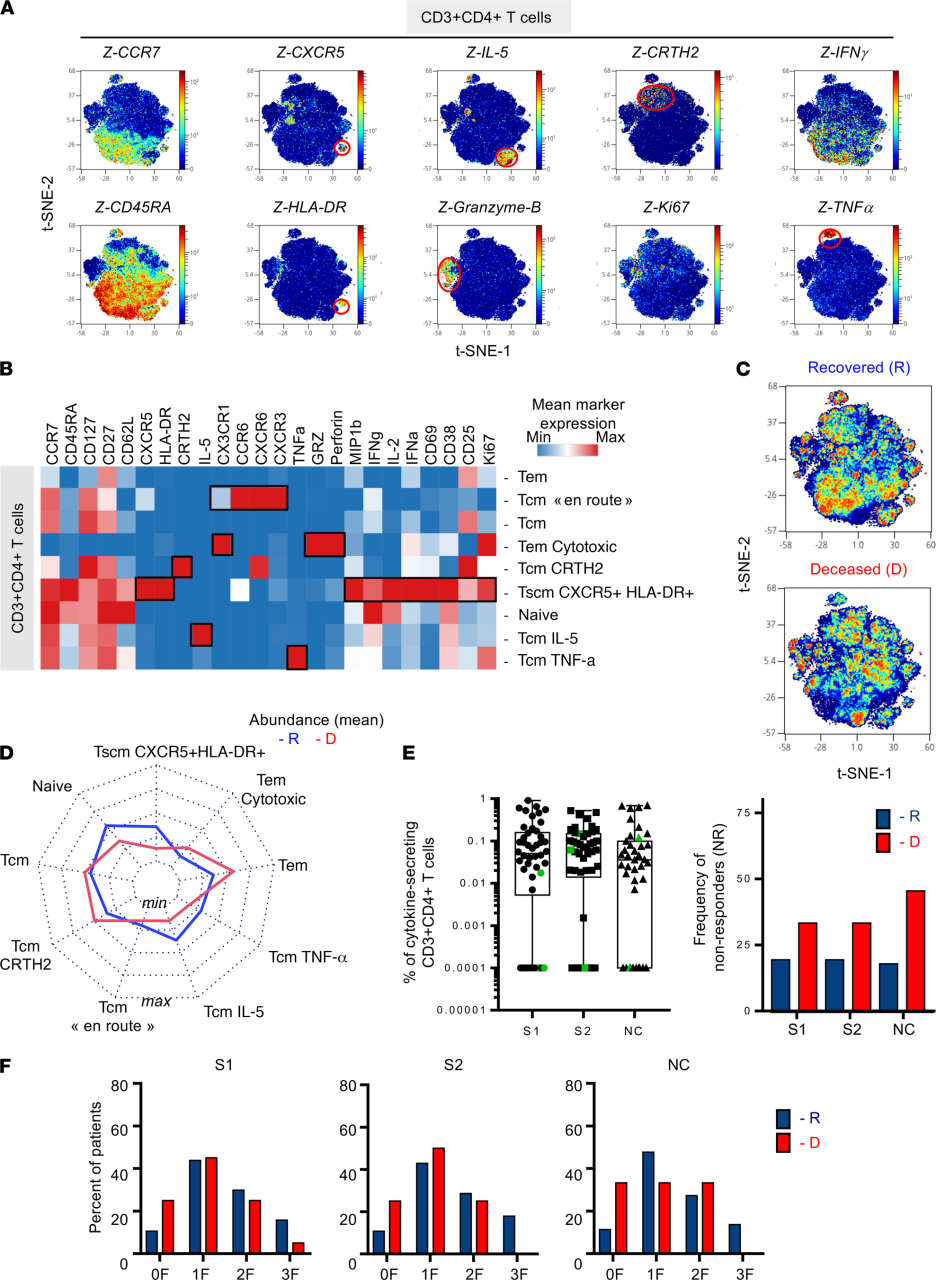
Increased frequencies of circulating polyfunctional CXCR5^+^HLA-DR^+^CD4^+^ T cells and Tem subsets are associated with survival in patients critically infected with COVID-19. PBMCs from 21 patients critically ill with COVID-19 were incubated with brefeldin A (16 hours) and stained using multiparametric mass cytometry panel 2 (*n* = 21, R = 12, D = 9). (**A**) CD3^+^CD4^+^ T cells (20,000 subsampling events) were randomly taken for unsupervised cluster using FlowSOM. Density plot t-SNE represents the expression of indicated markers. (**B**) Heatmap representation of mean signal intensity of each marker in CD3^+^CD4^+^T cells. (**C**) Density plot t-SNE representing abundance of events using concatenated files of 12 R and 9 D patients. (**D**) Radar representing mean (min/max normalized) abundances of CD3^+^CD4^+^ T cell subsets in 12 R (blue) and 9 D (red) patients. Multiple Mann-Whitney *U* test using Benjamini, Krieger, and Yekutieli FDR correction was performed, with significance set at *q* < 0.05. (**E**) SARS-CoV-2–specific T cell responses were measured in PBMCs from 46 ICU patients on day 15 ± 0.85 (mean ± SEM) after symptoms onset. PBMCs were stimulated for 16 hours with SARS-CoV-2 overlapping peptides: S1, S2, and NC. The frequency of specific CD4^+^T cells (Boolean gating of IFN-γ, IL-2, and TNF-α) is represented with box-and-whisker plots (min to max) after background subtraction according to background control (left). Color-coded (green) symbols represent individuals that were under immunosuppressive treatment when SARS-CoV-2–specific responses were studied. The frequency of nonresponders (with *<*0.005% cytokine-secreting CD3^+^CD4^+^ T cells) is represented among R (blue; S1 and S2 *n* = 31; NC *n* = 28) and D (red; S1 and S2 *n* = 15; NC *n* = 11) patients (right). (**F**) Frequency of patients with cells producing cytokines (0, 1, 2, 3 F) after stimulation in R (blue) and D patients (red). χ^2^ test did not show significance. R, recovered; D, deceased; F, function; Tem, effector memory T cell; tSNE, t-distributed stochastic neighbor embedding; NC, nucleocapsid.

To evaluate the expression of effector molecules by CD3^+^CD4^+^ T cells, PBMCs (*n* = 21) were incubated in vitro with brefeldin A for 16 hours prior to staining using mass cytometry panel 2 (Supplemental Table 2). According to unsupervised FlowSOM analyses, we identified 9 clusters of CD3^+^CD4^+^ T cells according to the expression of T cell differentiation surface markers, chemokine receptors, and effector molecules (Figure 2). Figure 2A represents density plots of all 9 clusters by t-SNE visualization. Several marker intensities were represented in each density plot and could be mapped based on CCR7 and CD45RA expression. Additional representation of the mean expression of markers by a heatmap allowed for an overview of the expression levels of these molecules (Figure 2B). We confirmed the identification of Tscm CXCR5^+^HLA-DR^+^ as observed in Figure 1B when using our mass cytometry panel 2. Interestingly, these cells secreted a panel of cytokines such as MIP-1β, IFN-γ, IL-2, and activation/proliferation markers, i.e., CD69, CD38, CD25, and Ki67 (Figure 1B). These results identify that these cells were an important source of cytokine production.

We revealed additional subsets within Tem and Tcm populations. Among Tcms (CCR7^+^ CD45RA^–^), we identified Tcm CRTH2, Tcm IL-5^+^ (Th2-like), Tcm “en route” (expressing CX3CR1, CCR6, CXCR6, and CXCR3, as major lung-homing receptors), and Tcm TNF-α^+^ cells (Figure 2, A and B). Among the Tem population, high levels of granzyme B and perforin expression were observed and associated with CX3CR1 expression. These results suggest the capacity of cytotoxic Tems to potentially migrate to inflammatory sites. As described in the literature, skewed T cell differentiation seems to occur in patients with COVID-19 with modified proportion and phenotype of Th1, Th2, Th17, and Tfh (19).

Visualization of concatenated files in density plot according to recovered and deceased patients showed significant differential abundance of several clusters (Figure 2C). Visualization of subpopulation abundances is shown in the radar chart (Figure 2D). Compared with ICU patients who died, ICU patients who recovered showed higher, but not significant, frequencies of naive cells and Tscm CXCR5^+^HLA-DR^+^ (multiple Mann-Whitney *U* test using Benjamini, Krieger, and Yekutieli FDR correction), with lower, but not significant, frequencies of Tems.

To better define antiviral CD4 functions, we measured antigen-specific effector CD4^+^ T cells by intracellular cytokine staining (ICS) flow cytometry for IFN-γ, IL-2, and/or TNF-α after in vitro stimulation with S1-, S2-, and NC-overlapping 15-mer peptides (Figure 2E). S1-, S2-, and NC-specific CD4^+^ T cells were heterogeneous among ICU patients (S1, S2 *n* = 46; NC *n* = 39; Figure 2E, left). Approximately 20% of patients who recovered did not display S1-, S2-, and NC-specific CD4^+^ T cells (nonresponders; Figure 2E, right). This proportion was approximately 30%–40% in deceased patients. The ICS analysis showed mostly monofunctional (mainly IFN-γ^+^) and bifunctional responses (IFN-γ^+^TNF-α; Figure 2F). Although there was a tendency of a higher frequency of nonresponders to S1-, S2-, and NC-overlapping peptides in deceased patients, we did not observe any significant differences. Additional measurement of serum levels of RBD, S1, S2 and NC-specific IgM, IgG and IgA (Supplemental Figure 2A) did not allow us to distinguish between deceased patients and recovered patients (Supplemental Figure 2B).

Therefore, CD4^+^ T cell differentiation/functional profiles as well as antigen-specific CD4^+^ T cell responses showed marginal modification at the critical phase of SARS-CoV-2 infection.

### Surviving patients with COVID-19 had increased levels of PD-L1^+^CXCR3^+^CD8^+^ Teffs and CXCR5^+^HLA-DR^+^CD8^+^ Tscms.

**Figure 3:**
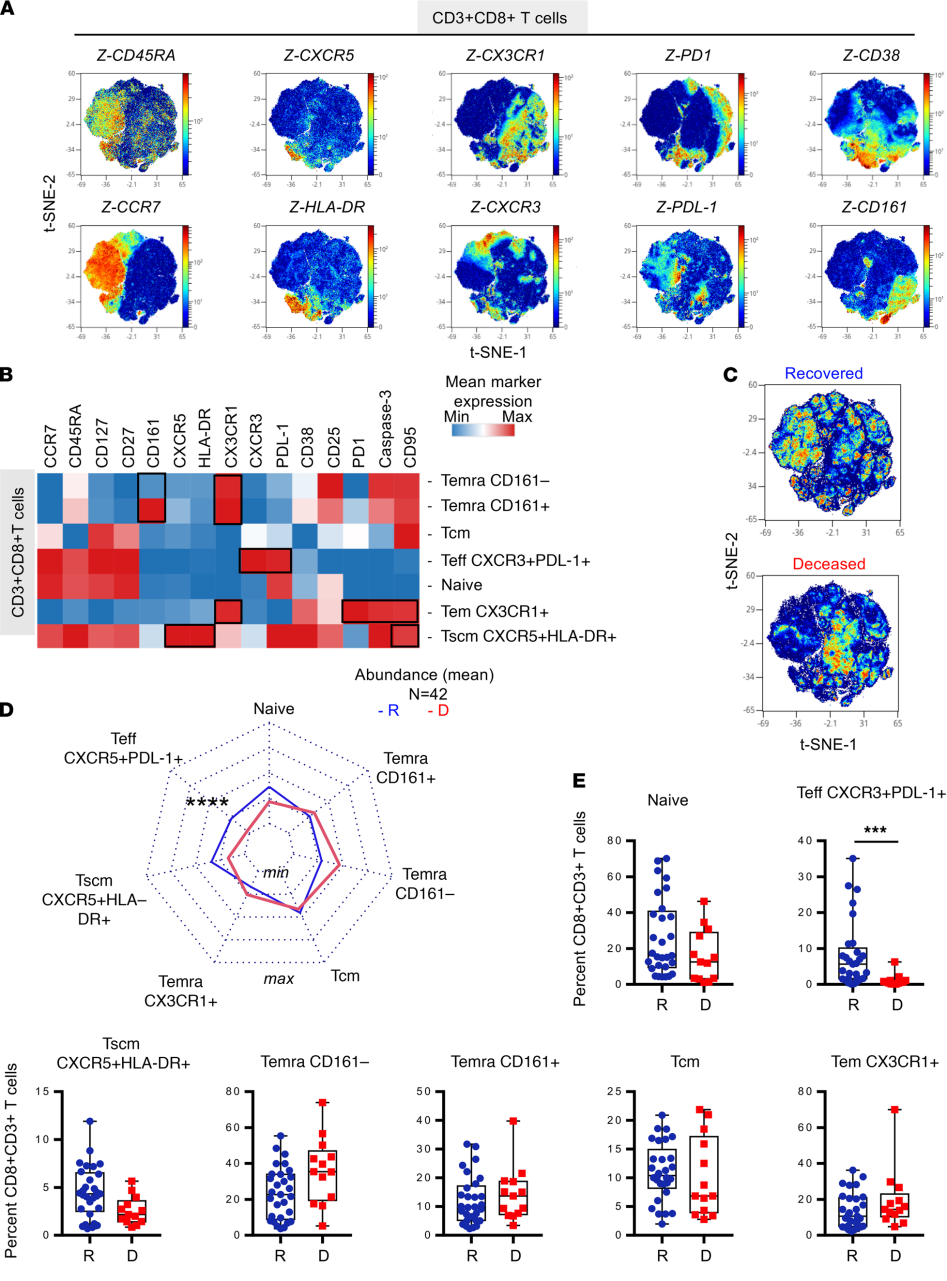
Surviving patients with COVID-19 have increased levels of PD-L1^+^CXCR3^+^CD8^+^ Teffs and CXCR5^+^HLA-DR^+^CD8^+^ Tscms. PBMC samples were collected to assess T cell phenotypes (*n* = 42, R = 29, D = 13) using mass cytometry panel 1 (Supplemental Table 1). CD3^+^CD8^+^ T cells (50,000 events) were randomly taken among sample for unsupervised cluster using FlowSOM. (**A**) Density plot t-SNE representing the expression of indicated markers. (**B**) Heatmap representation of mean signal intensity of each marker in identified CD3^+^CD8^+^T cell population. (**C**) Density plot t-SNE representing abundance of events using concatenated files of 29 R and 13 D patients. (**D**) Radar representing mean (min/max normalized) abundance of CD3^+^CD8^+^T cell subsets in 29 R (blue) and 13 D (red) patients. (**E**) Box-and-whisker plots with min and max of CD3^+^CD8^+^T cell subset abundances in 29 R (blue) and 13 D (red) patients. All points are shown. Multiple Mann-Whitney *U* test using Benjamini, Krieger, and Yekutieli FDR correction was performed, with significance set at ***P <.001. Teffs, effector T cells; Tscm, stem cell memory T cell; R, recovered; D, deceased.

A similar approach was used to analyze CD3^+^CD8^+^ T cell differentiation and determine their functional profile (Figure 1A and Supplemental Figure 1). According to unsupervised clustering and using density plot representation, we first observed the distribution of 15 markers including CCR7, CD45RA, CXCR5, HLA-DR, CX3CR1, CXCR3, PD-L1, PD-1, CD38, and CD161, as depicted in Figure 3, A and B. CCR7 and CD45RA mapping and indicated markers allowed us to label T cells subsets, according to the literature nomenclature, as naive, Tem, Tscm, Tcm, and Temra subsets (Supplemental Figure 1). Seven major clusters were then defined and represented in a heatmap (Figure 3B). As represented in Figure 3B, among CD3^+^CD8^+^ T cells, we defined clusters of cells as naive (CCR7^+^CD45^+^CD127^+^CD27^+^) and CXCR5^+^HLA-DR^+^CD8^+^ T cells (CCR7^+^CD45RA^+^ also expressing CD27, CD95, PD-L1, CD38, and caspase-3). According to the literature and similar to CD4^+^ T cells, CD8^+^ CXCR5^+^HLA-DR^+^ T cells shared CCR7, CD45RA, CD27, CXCR3, and CD95 with Tscms, as previously described in patients with HIV (22). Among CD3^+^CD8^+^ Teffs, we identified Tem CX3CR1 (also expressing exhaustion markers PD-1, caspase-3, and CD95) and a population of Teff PD-L1^+^CXCR3^+^ that remained CCR7^+^CD45RA^+^ and did not express CD95 (Figure 3B). Temra CD161^+^ and CD161^–^ CD8^+^ T cells (CCR7^–^ CD45^int^ also expressing CX3CR1) as well as 1 subset of Tcm were also identified. Visualization of concatenated files in density plot representation according to recovered and deceased patients showed significant differential abundance of several clusters (Figure 3C). Statistical analyses are shown in radar charts and box plots (Figure 3, D and E). Using multiple Mann-Whitney *U* test with Benjamini, Krieger, and Yekutieli FDR correction, we show that ICU patients who recovered compared with those who died, had higher frequencies of naive T cells (adjusted *P* = 0.07) and Tscm CXCR5^+^HLA-DR^+^ (adjusted *P* = 0.07) and a lower frequency of Temra CD161^–^ (*P* < 0.05). The main difference was observed in the higher frequencies of Teff PD-L1^+^CXCR3^+^ (adjusted *P* = 0.0006) in ICU patients who recovered compared with those who died. According to the literature, CXCR3^+^CD8^+^ T cells have been identified as a biomarker that is associated with survival in melanoma patients with stage III disease (28), suggesting a potential role of this marker in SARS-CoV-2–infected patient survival.

In conclusion, similarly to the CD4^+^ T cell compartment, unbalanced differentiation of CD8^+^ T cells allowed us to distinguish between ICU patients who recovered and those who died in critical cases of SARS-CoV-2 infection. We identified a subpopulation of CD8^+^ T cells expressing PD-L1^+^CXCR3^+^ that was significantly underrepresented in deceased patients and could have a potential role in disease control.

### Abundance of polyfunctional PD-L1^+^CXCR3^+^CD8^+^ T cells and NC-specific, cytokine-producing T cells defined survival versus fatal outcome after critical SARS-CoV-2 infection.

**Figure 4:**
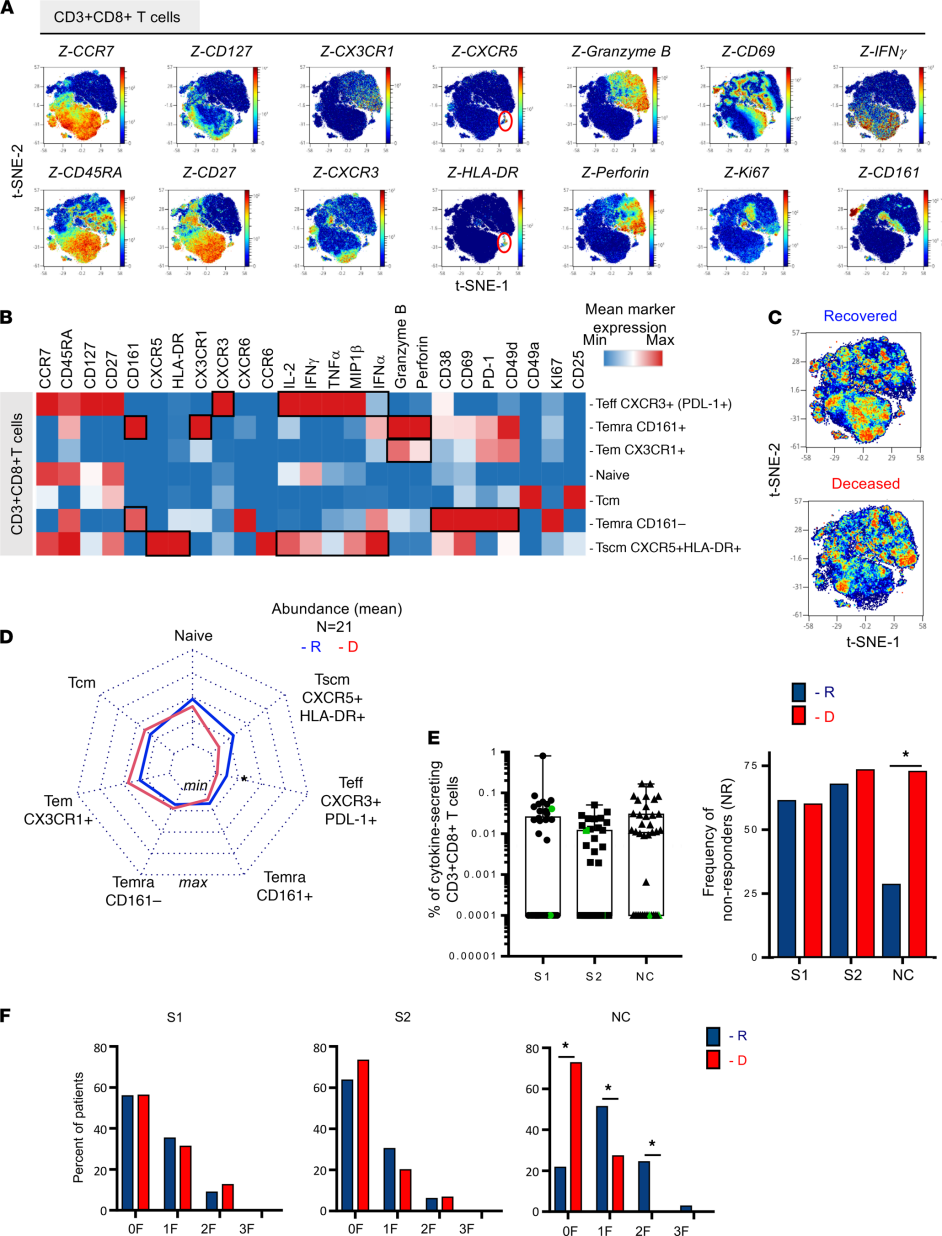
Abundance of polyfunctional PD-L1^+^CXCR3^+^CD8^+^ T cells and NC-specific, cytokine-producing T cells define survival vs. fatal outcome after critical SARS-CoV-2 infection. PBMCs from critical patients with COVID-19 were incubated with brefeldin A (16 h) and stained using multiparametric mass cytometry panel 2 (*n* = 21, R = 12, D = 9). (**A**) CD3^+^CD8^+^ T cell (20,000 events) were randomly taken among sample for unsupervised cluster using FlowSOM. Density plot t-SNE representing the expression of indicated markers. (**B**) Heatmap representation of mean signal intensity of each marker in CD3^+^CD8^+^T cell subsets. (**C**) Density plot t-SNE representing abundance of events using concatenated files of 12 R and 9 D patients. (**D**) Radar representing mean (min/max normalized) abundances of CD3^+^CD4^+^T cell subsets in 12 R (blue) and 9 D (red) patients. Multiple Mann-Whitney *U* test using Benjamini, Krieger, and Yekutieli FDR correction was performed, with significance set at adjusted *P <.*05. (**E**) SARS-CoV-2–specific T cell responses were measured in PBMCs from 46 ICU patients on day 15 ± 0.85 (mean ± SEM) after symptom onset. PBMCs were stimulated for 16 hours with SARS-CoV-2 overlapping 15-mer peptides (S1, S2, and NC). The frequency of specific CD8^+^ T cells (Boolean gating of IFN-γ, IL-2, and/or TNF-α) is represented with box-and-whisker plots (min to max) after background subtraction according to background control (left). Color-code (green) symbols represent individuals that were under immunosuppressive treatment when SARS-CoV-2–specific responses were studied. Individuals were considered responders when the frequency of cytokines produced was >0.005% of CD3^+^CD8^+^ cells. The frequency of nonresponders is represented among R (blue, S1 and S2: *n* = 31; NC *n* = 28) and D (red, S1 and S2 *n* = 15; NC *n* = 11) patients (right). χ^2^ test was performed, with significance set at *P < 0.05. (**F**) Frequency of patients with detectable cells producing cytokines (0, 1, 2, 3 F) after stimulation in R (blue) and D patients (red). χ^2^ test was performed, with significance set at *P < 0.05. R, recovered; D, deceased; NC, nucleocapsid; F, function.

To evaluate the expression of effector molecules by CD3^+^CD8^+^ T cells, PBMCs (*n* = 21) were incubated in vitro with brefeldin A for 16 hours prior to staining using mass cytometry panel 2 (Supplemental Table 2). According to unsupervised FlowSOM analysis, we identified 7 major clusters of CD3^+^CD8^+^ T cells assigning T cell differentiation surface markers, chemokine receptor, and effector molecules expression (Figure 4). Figure 4A represents the density plot of all 7 clusters using t-SNE visualization. Intensity and mapping of several markers including CCR7 and CD45RA as well as chemokine receptors, activation/proliferation, and differentiation markers are depicted in Figure 4A. An additional heatmap allowed for an overview of the expression levels of these molecules (Figure 4B). We confirmed the identification of 7 major clusters of CD3^+^CD8^+^ T cells similar to Figure 3. Namely, naive, Tscm CXCR5^+^HLA-DR^+^, Teff PD-L1^+^CXCR3^+^, Tcm, Temra CD161^+^, and Temra CD161^–^ populations were identified among CD3^+^CD8^+^ T cells.

Interestingly, Teff PD-L1^+^CXCR3^+^ remained the most polyfunctional cells with the highest production levels of multiple cytokines including MIP-1β, IFN-γ, IL-2, and TNF-α followed by Tscm CXCR5^+^HLA-DR^+^, which also expressed MIP-1β, IFN-γ, IL-2, TNF-α, and IFN-α. These 2 subsets of CD8^+^ T cells remain significantly higher in patients who survived, as shown in the radar charts (Figure 4D). Their frequencies remained similar to the ones observed in Figure 3E. Tem CX3CR1 CD8^+^ T cells expressed lung-homing markers, exhaustion markers PD-1, and cytotoxic molecules (granzyme B and perforin). However, these expressions were lower compared with the Temra CD161^+^ cells as represented in the heatmap (Figure 4B). These results suggest that these 2 populations of relatively higher abundance in deceased patients might be harmful. Temra CD161^+^ also expressed CX3CR1, a homing marker to the lung, suggesting a role of this population during SARS-CoV-2 infection.

We also measured antigen-specific CD8^+^ Teffs by ICS flow cytometry for IFN-γ, IL-2, and/or TNF-α after in vitro stimulation with S1-, S2-, and NC-overlapping 15-mer peptides (Figure 4, E and F). S1-, S2-, and NC-specific CD8^+^ T cells were very heterogeneous among ICU patients (S1, S2 *n* = 46; NC *n* = 39). Approximately more than 50% of patients did not display spike-specific CD8^+^ T cells (nonresponders; Figure 4E). Interestingly, the proportion of individuals without detectable NC-specific CD8^+^ T cells was significantly higher in patients who deceased (73% nonresponders) compared with patients who recovered (25% nonresponders). The ICS analysis showed mostly monofunctional responses (mainly IFN-γ^+^) and bifunctional response (IFN-γ^+^TNF-α). The lack of NC-specific CD8^+^ T cells was marked for all 3 functions tested.

Altogether, we show that the low abundance of polyfunctional Teff PD-L1^+^CXCR3^+^ and Tscm CXCR5^+^HLA-DR^+^CD8^+^ T cells and the lack of NC-specific cytokine-producing CD8^+^ T cells were key features of patients with fatal outcome after critical SARS-CoV-2 infection.

### PD-L1^+^CXCR3^+^ and NC-specific CD8^+^ T cell frequencies predicted fatal outcome of patients after critical SARS-CoV-2 infection.

**Figure 5:**
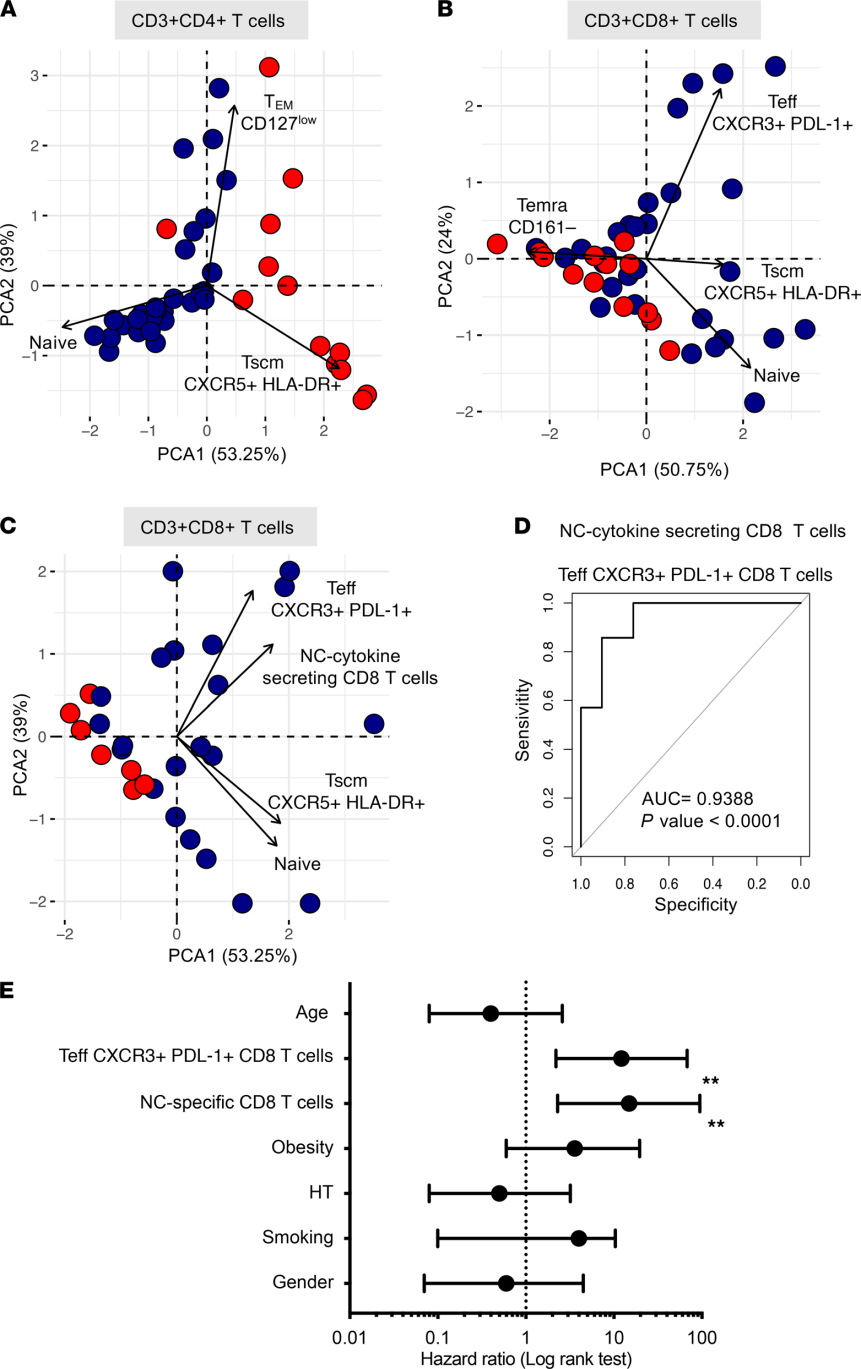
PCA of T cell frequencies discriminate survival and decreased ICU patients after critical SARS-CoV-2 infection. PCA representations (R software) are as follows: (**A**) CD4^+^ T cell subset abundance and (**B**) CD8^+^ T cell subset abundance as indicated, and (**C**) CD8^+^ T cell subsets as indicated and frequency of NC-specific CD8^+^ T cells. Color code indicates patients who recovered (blue) and patients who deceased (red). (**D**) ROC curve (R software) modeling the abundance of PD-L1^+^CXCR3^+^ Teffs and NC-specific CD8^+^ T cells in disease survival or death outcomes. AUC = 0.9388, *P* < 0.001. (**E**) Forest plots comparing HR (Mantel-Haenszel) for death in 28 patients according to the abundance of PD-L1^+^CXCR3^+^ Teffs and NC-specific CD8^+^ T cells. Log rank (Mantel–Cox) test was used to compare HR between groups, with significance defined by a **P < 0.001. PCA, principal component analysis; ROC, receiver operating characteristic; NC, nucleocapsid; Teffs, effector T cells.

We used principal component analysis (PCA) to visualize and better understand the underlying structure of the data in an unsupervised way, by reducing multidimensional data sets in a 2-dimensional representation. This allowed us to take into account the similarities between subjects to have a robust informative viewpoint while preserving a high percentage of the variation of the initial data set, i.e., CD4^+^ and CD8^+^ T cell subsets (*n* = 42; Figure 5, A and B, respectively) and CD8^+^ T cells plus NC-specific CD8^+^ T cells (*n* = 28; Figure 5C). In Figure 5, A and B, the first 2 principal components allowed us to efficiently distinguish 2 groups as recovered patients and deceased patients. Analysis of CD4^+^ and CD8^+^ data sets caught approximatively 92% and 75%, respectively, of the variation of the initial information, thus allowing for conserving most information. We then added to the PCA the information on NC-specific CD8^+^ T cells (92% data set used) to distinguish recovered patients from deceased patients. Using PCA, we demonstrated that balance in the T cell compartment during SARS-CoV-2 infection allowed for the differentiation of clusters of patients who recovered and patients with fatal outcome. Among all subpopulations, the most significant changes were observed in Teff PD-L1^+^CXCR3^+^ and NC-specific CD8^+^ T cells. Hence, we used logistic regression analysis to consider whether Teff PD-L1^+^CXCR3^+^ and NC-specific CD8^+^ T cells might suppose the survival or fatal outcomes. The receiver operating characteristics (ROC) curve showed that Teff PD-L1^+^CXCR3^+^ and NC-specific CD8^+^ T cells were the most accurate prognosticator of fatal outcomes (AUC = 0.9354, *P* < 0.0001; Figure 5D). As shown in Figure 5E, we segregated patients into 2 groups — those who recovered and those who died from COVID-19 — and compared their relative risk using a Cox proportional hazard model with other confounding factors, including age, gender, hypertension (HT), and obesity and the 2 main features of adaptive T cell immune responses to the virus, i.e., the proportion of NC cytokine-secreting CD8^+^ T cells and Teff CXCR3^+^PD-L1^+^CD8^+^ T cells. Again, patients who had a lower abundance of NC cytokine-secreting CD8^+^ T cells and Teff CXCR3^+^PD-L1^+^CD8^+^ T cells were at a higher risk of death. The HR (Mantel-Haenszel) of NC cytokine-secreting CD8^+^ T cells and Teff CXCR3^+^PD-L1^+^CD8^+^ T cells was 14.9 and 12.2, respectively. The HR calculated for confounding factors such as age, smoking, obesity, and HT was not statistically significant using this model.

In conclusion, these 2 parameters had correctly foreseen survival or death prognostics of patients after critical SARS-CoV-2 infection, reinforcing the important role of T cells during COVID-19 infection.

## Discussion

Our study focused on the analysis of T cell responses in ICU patients in critical condition to decipher the role of T cells in acute viral SARS-CoV-2 infection. Although a humoral response was detected in all patients, it did not allow us to distinguish patients who survived from those with fatal outcome. In contrast, a significant disequilibrium in the frequency of CD4^+^ and CD8^+^ subsets was found to be characteristic for patients who recovered or died during ICU hospitalization.

Laing et al. (29) compared the immunological signature in asymptomatic/mild COVID-19 with that of healthy controls. It is evident that disorder in immune parameters could be major when comparing “disease” to “health” status. Laing et al. (29) observed disproportionate depletions of CD4^+^ Th17 and Th1 cells and Tregs in patients with COVID-19 compared with healthy adults. According to the literature, during SARS-CoV-2 infection, there is an increased proportion of cytotoxic Tfhs and cytotoxic Th cells (CD4-CTLs) responding to SARS-CoV-2 and a reduced proportion of SARS-CoV-2–reactive Treg in hospitalized patients compared with those in the ICU (20).

In our study, the proportion of CD4^+^ T cell subsets was found to be significantly different with respect to disease outcome in severely infected patients when comparing deceased patients with recovered ones. We also observed that among CD4^+^ T cells, a higher abundance of naive T cells and polyfunctional Tscms CXCR5^+^HLA-DR^+^ and a lower abundance of Tems, including those expressing granzyme B and perforin, were observed in patients who recovered. Tscm CXCR5^+^HLA-DR^+^ shared Tscm markers such as CCR7, CD45RA, CD27, CXCR3, and CD95, as previously described in HIV infection (22). Polyfunctionality of these cells in regard to major Th1 cytokine production (MIP-1β, IFN-γ, IL-2) and expression of activation/proliferation markers, i.e., CD69, CD38, CD25, and Ki67, suggest a potential central role in the control of infection. CD4^+^ Tscms are efficiently induced after yellow fever vaccination and persist for decades (30). Different strategies have been explored to expand Tscms in vitro for tumor therapy because they can proliferate and survive vigorously under the continuous stimulation of tumor antigen (31). Inversely to Tscm upregulation in recovered patients, CX3CR1-expressing Tems were more abundant in deceased patients. The expression of chemokine receptors and cytotoxic molecules (granzyme B and perforin) by Tem suggest that they might migrate to tissue inflammation and induce damage, according to the literature (32).

The Tcm subset expressed CXCR3^+^, CX3CR1^+^, CXCR6, and CCR6, which we named “en route” because of the high level of expression of multiple chemokine receptors. Among these homing receptors, CXCR6 is a chemokine receptor that allows cell homing to the lung. However, it has been shown in both tuberculosis and influenza mouse models that CXCR6 deficiency does not affect the capacity of cells to migrate into the lung but is associated with an improved control of the infection (33). We also observed that SARS-CoV-2–specific CD4^+^ T cell responses, although undetectable in the blood of a fraction of ICU patients, did not discriminate between recovered patients and deceased patients.

Among CD8^+^ T cells, the abundance of polyfunctional PD-L1^+^CXCR3^+^ T cells and Tscm CXCR5^+^HLA-DR^+^ and significantly higher responders to NC antigen allowed us to distinguish between recovered patients and deceased patients. CXCR5^+^HLA-DR^+^CD8^+^ T cell populations shared phenotypic profiles of Tscm, as described in cancer and in chronic viral infection such as HIV (22). Tscms exhibit characteristics of conventional memory T cells. In patients with HPV-associated cancer, CD8^+^ Tscms were found to have long-term antitumor function both in vivo and in vitro (31). The T cell receptor rearrangement excision circles are similar in Tcm and Tem, suggesting that they underwent multiple division (34). Of note, we also found Ki67 expression on Tscm CXCR5^+^HLADR^+^CD8^+^ T cells compared with other CD8^+^ T cell subsets from patients with COVID-19. The role of these cells in infection has been shown to be more efficient than their CXCR5^–^ counterpart for viral load control (35). Tscms are detected in both CD4^+^ and CD8^+^ T cell populations of mice (36) and nonhuman primates and humans (37). They also have been proposed as a weapon in cancer immunotherapies. According to the literature and our observation of polyfunctionality of this population, we suggest that the Tscm CXCR5^+^HLA-DR^+^CD8^+^ T cells might have an importance for virus clearance in COVID-19.

Among CD8^+^ T cells, we also identified PD-L1^+^CXCR3^+^ T cells with a polyfunctional cytokine profile producing MIP-1β, IFN-γ, IL-2, and TNF-α. Increased IP-10 production induced by type 1 IFN in inflamed tissue could participate in the attraction of Teff PD-L1^+^CXCR3^+^CD8^+^ cells and thus participate in viral control. Their presence in recovered patients, compared with deceased patients, could be a signature of viral control. According to the literature, an increase in CXCR3^+^CD8^+^ T cells has been identified as a biomarker that is associated with survival in melanoma patients with stage III disease (28), suggesting a potential role of this marker in the survival of patients infected with SARS-CoV-2. In addition, a significantly lower percentage of CD8^+^ T cells directed against NC were observed in deceased patients. Spike-specific CD8^+^T cells were detectable in deceased patients but lacked polyfunctionality. ROC analysis validated that the lack of PD-L1^+^CXCR3^+^ Teffs and NC-specific CD8^+^ T cells correctly forecasted fatal disease outcome with a 93% accuracy.

Studies have demonstrated the important role of memory T cells in the adaptive immune response to viral infections (7, 38) and have pointed out that they can be either beneficial or detrimental with respect to tissue damage during COVID-19 infection (25). The potential protective role of NC-specific CD8^+^ T cell responses could be of importance in future vaccine design (39). Indeed, unlike spike protein, the internal NC protein is highly conserved among coronavirus strains, which might allow for a cross-protection between strains, is abundantly expressed during infection, and is highly immunogenic (40, 41). Indeed, spike protein is more likely to be subjected to a pressure of selection. We are facing the emergence of new SARS-CoV-2 variants, originally discovered in Brazil, United Kingdom, and South Africa, with mutations on the spike protein, which makes the virus up to 71% more contagious (42). The strategy to use NC antigen in vaccine design could participate in the control of emerging SARS-CoV-2 variants. Further studies are necessary to evaluate the potential role of these cell populations as surrogate markers for viral control. Combining the knowledge from T cell immunology and induction of polyfunctional effector and memory cells will be beneficial for future vaccine design and its immunomonitoring.

## Methods

### Study participants.

A total of 56 adult patients with COVID-19 referred to the Department of Internal Medicine 2, Department of Infectious Diseases and ICUs, Pitié Salpêtrière Hospital, Paris, were included in the study between March 2020 and May 2020. The diagnosis of COVID-19 relied on SARS-CoV-2 carriage in the nasopharyngeal swab, as confirmed by real-time reverse transcription PCR analysis. Demographic and clinical characteristics are detailed in Table 1. One third of the patients died from COVID-19. Patient age ranged from 25 to 75 years (median: 55 years, IQR 48–62), with 12.7% of patients older than 65 years of age. Concerning immune traits, patients presented with general lymphopenia and granulocytosis, but no significant differences were observed between survivors and nonsurvivors. Delay between first symptom appearance and ICU admission has been found to be homogeneous with a median at 9 days (IQR 7–9). Sampling was performed a few days after admission (median 6 days after admission, IQR 4–7 days after admission for specific T cell responses measurement, and slightly before for cell phenotyping). The time between symptom onset and death was a median of 21 days (IQR 16–51). The interval between phenotyping and death was 8 ± 4 days (median ± SEM) after immunomonitoring antigen-specific T cell responses.

### Blood sample preparation.

For all patients, whole blood was collected in acid citrate dextrose tubes. PBMCs were isolated by density-gradient sedimentation using lymphocyte separation medium (Eurobio). Isolated PBMCs were cryopreserved in FBS (Dutscher) containing 10% DMSO and stored at –150°C. PBMCs were used for the measurement of SARS-CoV-2 antigen-specific T cells and mass spectrometry staining. Serum was prepared from whole blood collected in tubes without coagulant and stored at –80°C for anti-SARS-CoV-2 antibody measurement.

### Serum antibody dosage.

The presence of serum antibodies, specific for viral antigen, was determined with the Maverick SARS-CoV-2 Multi-Antigen Serology Panel (Genalyte) following the manufacturer’s instructions. This technology has been designed to detect antibodies specific for the 5 SARS-CoV-2 antigens (NC, spike RBD, full-length spike S1+S2, spike S1 and spike S2 subunits), immobilized on a chip, within a multiplex format based on photonic ring resonance technology. It detects and measures changes in resonance when antibodies bind to their respective antigens. Threshold values for positivity were set by the manufacturer (43, 44).

### Antigen-specific T cells and ICS.

PepMIX SARS-CoV-2 peptide pools (JPT Peptide Technologies) corresponding to 15-mer overlapping peptides of the NC (102 peptides), spike subunit 1 (S1, 157 peptides), and spike subunit 2 (S2, 158 peptides) were used to measure SARS-CoV-2–specific T cell responses. PBMCs were thawed and rested for 5 hours in complete medium (RPMI 1640 medium added with 10% bovine serum, Dutscher; 1% L-glutamine, 1% streptomycin/neomycin, 1% sodium pyruvate, and 1% nonessential amino acid, Gibco). After resting, cells were washed and distributed in round bottom 96-well plates. Stimulation was performed with 1.5 μg/ml of S1, S2, or NC peptide pools. Medium containing DMSO was used as unstimulated control, and human Dynabeads CD3/CD28 (Gibco) was used for positive control. After 1 hour, brefeldin A (MilliporeSigma) was added at a final concentration of 10 μg/ml. Cells were cultured for an additional 15 hours, before flow cytometry ICS, as follow: live and dead staining was performed using live/dead fixable kit (Molecular Probes), followed by surface staining at 4°C using anti–CD3-APC-H7 (clone SP 34-2), anti–CD8-FITC (clone SK1) antibodies (BD Biosciences), and anti–CD4 BV650 (clone OKT4, Biolegend). PBMCs were then washed and fixed using the Fixation/Permeabilization Kit Solution (BD Biosciences). Anti–TNF-α-PE-Cy7 (MAb11), IFN-γ-AF700 (clone B27), CD3-APC-H7 (clone SP 34-2; BD Biosciences), and anti-IL-2-APC (clone N7.48 A; Miltenyi Biotec) were used for ICS. Cells were then washed and as described in chronic viral infection as described in cancer and HIV Fortessa X20 (BD Biosciences). Live events were analyzed by Boolean combination gating with FlowJo software (Tree Star Inc.). Background cytokine responses detected in negative controls were subtracted from those detected in stimulated samples.

### Mass cytometer staining.

For the PBMC phenotyping panel 1 (Supplemental Table 1), cells were thawed and rested for 1 hour in complete medium. For the PBMC functional characterization panel 2 (Supplemental Table 2), PBMCs were thawed and rested for 5 hours in complete medium before brefeldin A (10 μg/ml) was added for an overnight incubation. PBMCs (5 to 10 million) were used for the staining. Cell viability was evaluated with a cisplatin staining (Fluidigm) before blocking unspecific staining with Human Fc block (BD Biosciences). After surface staining, cells were washed with Maxpar Cell Staining Buffer (Fluidigm) and then fixed and permeabilized with the Transcription Factor Buffer Set (BD Biosciences). PBMCs were resuspended in heparin solution before adding the intracellular antibody mix. Finally, iridium staining was performed in PBS 2% PFA at 4°C overnight. Cells were then kept at 80°C for 1 to 3 weeks. After thawing, stained PBMCs were consecutively washed with PBS, Maxpar Cell Acquisition Solution (Fluidigm), and deionized water. Calibration beads in EDTA (Fluidigm) were added before the acquisition with a Helios at the CyPS.

### Mass cytometry analysis.

After sample acquisition, data from each sample were normalized (mass cytometer, software version 6.7.1014, Fuidigm). A quality control step consisting of checking the number of cell events and marker signal in comparison with an internal control was then performed. Data were then cleaned based on beads, Barium/Cesium contamination, doublet, and dead cell removal to keep only CD45^+^ cells. CD3^+^CD4^+^ T cells and CD3^+^CD8^+^ T cells (Supplemental Figure 1A) were selected prior to analysis using OMIQ (https://www.omiq.ai/). FlowSOM algorithm automatically split CD4^+^ T cell and CD8^+^ T cell populations into major clusters identified in regard to CCR7 and CD45RA (Supplemental Figure 1B). Cell clusters were labeled based on CD45RA/CCR7 expression. OMIQ platform was used to display Opt-SNE, FlowSOM analyses, and heatmap representations.

### Statistics.

PCA and ROC curve were performed using FactoMiner/FactoExtra and fmsb and pROC R packages, respectively. The combination of multiple parameters and AUC determination for the ROC models were performed by binomial generalized linear model. Mann-Whitney *U* tests were used when comparing abundance of cell populations in deceased versus recovered patients. Adjusted *P* values for multiple Mann Whitney *U* test procedures were generated including Benjamini, Krieger, and Yekutieli procedures for the control of the FDR. Corrected *P* values of less than 0.05 were considered statistically significant. Statistical analyses and graph representation were performed using either GraphPad Prism 9 Software or R.

### Study approval.

The study was conducted in accordance with the Declaration of Helsinki and the International Conference on Harmonization Good Clinical Practice guidelines and approved by the relevant regulatory and the ethics committee of AP-HP. All patients gave informed consent. The study was registered and approved by the local ethical committee of Sorbonne-Université/AP-HP for ICU patients (CER-2020-31).

## Author contributions

BC and CC designed the study, and PR and LA designed and performed flow cytometry and mass cytometry experiments with the support of AC and NG. CP and MM performed antibody dosage. LA performed flow cytometry analysis, and BC and PR performed mass cytometry data analyses. PR, LA, KD, CP, and OB participated in biobanking. PQ was responsible for clinical data mining and analysis. MM, CEL, GG, and AG provided patient sample access. BC and CC provided financial support. BC, CC, LA, and PR wrote the manuscript. All authors contributed in reviewing the manuscript. First authorship order was based on their contribution to the work.

## Supplementary Material

Supplemental data

## Figures and Tables

**Table 1:**
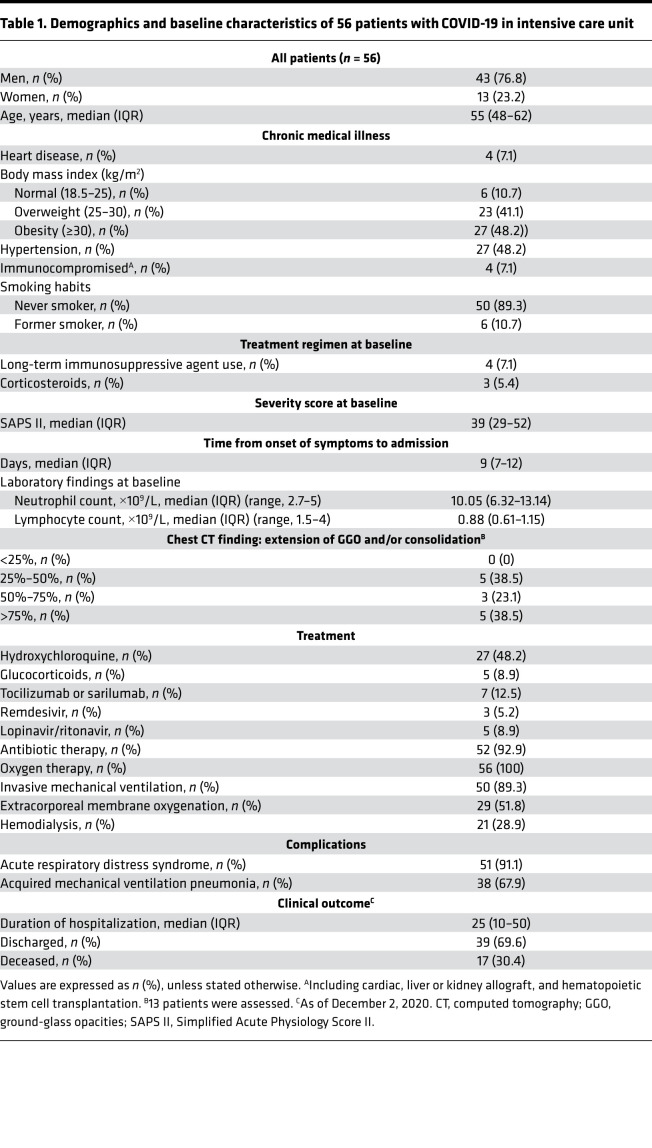
Demographics and baseline characteristics of 56 patients with COVID-19 in intensive care unit

## Version 1 07/20/2021

In-Press Preview

## Version 2 09/22/2021

Electronic publication
