# Supplementary material for: CD8+PD-L1+CXCR3+ polyfunctional T cell abundances are associated with survival in critical SARS-CoV-2–infected patients
Source: JCI Insight. 2021 Sep 22;6(18):e151571. doi: 10.1172/jci.insight.151571 (PMC8492305; doi:10.1172/jci.insight.151571)
Supplement: Supplemental data [file jciinsight-6-151571-s048.pdf]

151571-INS-RG1

Nucleocapsid-specific and PD-L1+CXCR3+ CD8 polyfuntional T-cell abundances are associated with survival of critical SARS-CoV2-infected patients

Lucille Adam et al.

Supplementary Figures

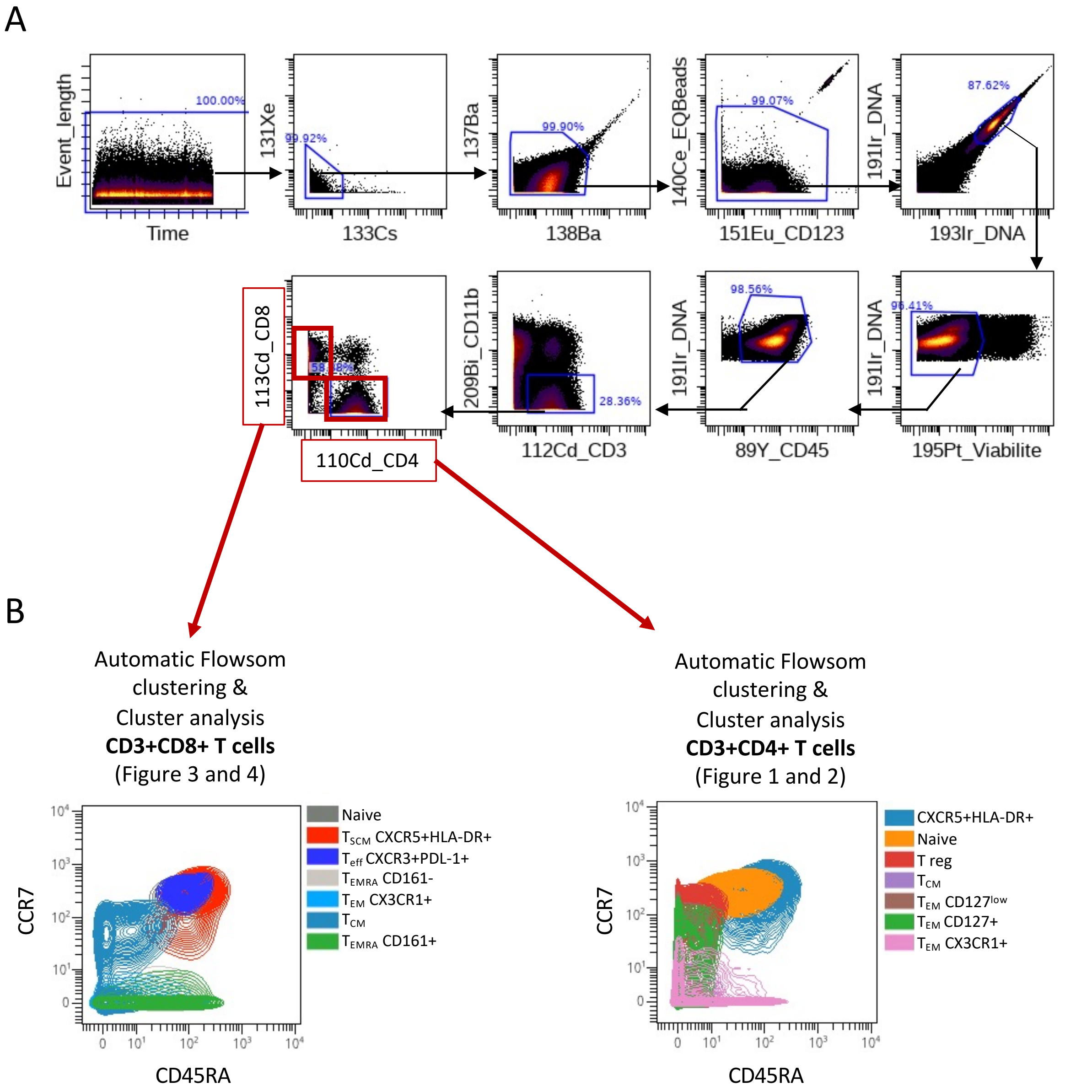

Supplementary Figure S1

Supplementary figure S1: Gating strategies. Representation of pipeline analysis of mass-cytometry for panel #1 and Panel #2. Each FlowSOM clustering-defined CD8+ (left) or CD4+ (right) T-cell subsets were represented according to their expression of CCR7 and CD45RA.

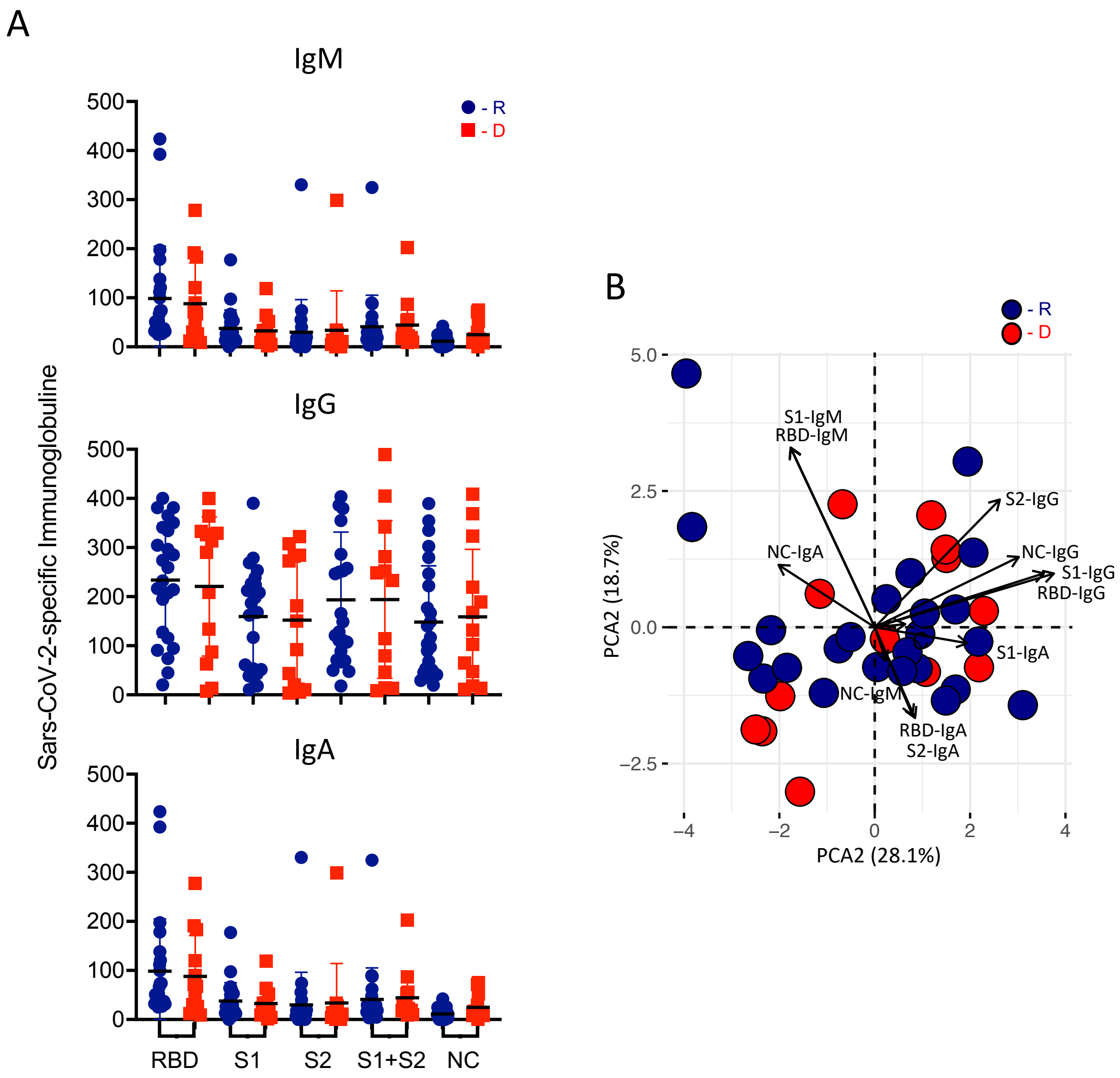

Supplementary Figure S2

Supplementary Figure S2: Humoral responses against RBD, Spike subunits 1 and 2, nucleocapsid of ICU SARS-CoV-2 infected patients. (A) Graphical representation of antibody titers according to isotype IgM, IgG and IgA and antigen specificity RBD, Spike subunit 1 (S1) and 2 (S2) and nucleocapsid (NC). (**B**) Principal component analysis (PCA) representation of anti-SARS-CoV-2 antibody titers. Color code indicate patients who recovered (blue) and patients who deceased (red).

151571-INS-RG1

Nucleocapsid-specific and PD-L1+CXCR3+ CD8 polyfunctional T-cell abundances are associated with survival of critical SARS-CoV2-infected patients

Lucille Adam et al.

Supplementary tables

Table S1: Mass cytometry Panel #1 T cell phenotyping

Channels	Markers	Clones	Suppliers
106Cd	CD11c	BU13	BD Biosciences
110Cd	CD14	M5E2	BD Biosciences
111Cd	CD4	SK3	Biolegend
112Cd	CD3	UCHT1	BD Biosciences
113Cd	CD8	SK1	Biolegend
114Cd	HLA-DR	L243	Biolegend
116Cd	CD19	HIB19	BD Biosciences
141Pr	CX3CR1	K1024-E1	Biolegend
142Nd	Caspase 3	D3E9	Biolegend
143Nd	CD95	DX2	Biolegend
144Nd	CD38	HIT2	Fluidigm
148Nd	CD16	3G8	Fluidigm
152Sm	CD45RA	HI100	BD Biosciences
155Gd	CD27	L128	Fluidigm
156Gd	CD183 (CXCR3)	G025H7	Fluidigm
159Tb	Foxp3	259D/C7	Fluidigm
161Dy	CD25 (IL-2R)	Hu107	R&D
162Dy	CD161	581	Biolegend
164Dy	CD185 (CXCR5)	51505	R&D
165Ho	CD127 (IL-7Ra)	A019D5	Fluidigm
167Er	CD197 (CCR7)	G043H7	Fluidigm
168Er	CD274 (PDL-1)	29R-2A3	Biolegend
173Yb	CD141	1A4	Fluidigm
174Yb	CD279 (PD-1)	EH12.2H7	Fluidigm
175Lu	CD66b	1H9	BD Biosciences
176Yb	CD56	NCAM16.2	Fluidigm
209Bi	CD11b (Mac-1)	ICRF44	Fluidigm
89Y	CD45	HI30	Fluidigm

Table S2: Mass cytometry Panel #2 for T cell function assessments

Channels	Markers	Clones	Suppliers
106Cd	CX3CR1	K1024-E1	Biolegend
111Cd	CD4	SK3	Biolegend
112Cd	CD3	UCHT1	BD Biosciences
113Cd	CD8	SK1	Biolegend
114Cd	HLA-DR	L243	Biolegend
116Cd	CD19	HIB19	BD Biosciences
141Pr	CD196 (CCR6)	G034E3	Fluidigm
143Nd	IL5	TRFK5	Fluidigm
144Nd	CD38	HIT2	Fluidigm
145Nd	CD62L	DREG-56	Biolegend
146Nd	TNFα	Mab11	Fluidigm
147Sm	CD49a	TS2/7	Biolegend
148Nd	Ki67	Ki67	Biolegend
149Sm	CD25 (IL-2R)	2A3	Fluidigm
150Nd	MIP1b	D12-1351	Fluidigm
151Eu	CD49d	9F10	Biolegend
152Sm	CD45RA	HI100	BD Biosciences
154Sm	CD69	FN50	Biolegend
155Gd	CD27	L128	Fluidigm
156Gd	CD183 (CXCR3)	G025H7	Fluidigm
159Tb	FoxP3	259D/C7	Fluidigm
160Gd	Tbet	4B10	Fluidigm
161Dy	CXCR6 (CD186)	K041E5	Biolegend
162Dy	CD161	581	Biolegend
163Dy	CRTH2	BM16	Fluidigm
164Dy	IL17A	N49-653	Fluidigm
165Ho	CD127 (IL-7Ra)	A019D5	Fluidigm
166Er	IL-2	MQ1-17H12	Fluidigm
167Er	CD197 (CCR7)	G043H7	Fluidigm
168Er	IFNγ	B27	Fluidigm
170Er	CD152 (CTLA-4)	14D3	Fluidigm
171Yb	CD185/CXCR5	51505	R&D
172Yb	IFNα	7N4-1	Fluidigm
173Yb	Granzyme B	GB11	Fluidigm
174Yb	CD279 (PD-1)	EH12.2H7	Fluidigm
175Lu	Perforin	B-D48	Fluidigm
176Yb	CD56	NCAM16.2	Fluidigm
209Bi	CD11b	ICRF44	Fluidigm
89Y	CD45	HI30	Fluidigm
